# Supplementary material for: Association of ABO blood group with COVID-19 severity, acute phase reactants and mortality
Source: PLoS One. 2021 Dec 14;16(12):e0261432. doi: 10.1371/journal.pone.0261432 (PMC8670663; doi:10.1371/journal.pone.0261432)
Supplement: Attachment — Submitted filename: response to reviewers.docx [file pone.0261432.s002.docx]

Dear editors and reviewers

Thank you for taking out time to comment on this manuscript. Your comments were valuable in making this manuscript better.

Point to point response

Reviewer 1

Issue 1: Many confounding factors would affect the clinical outcome (severity and mortality), such as comorbidities and medical treatment. Therefore, to minimize confounding bias, a multivariate analysis which includes variables of blood groups, comorbidities, and treatment should be performed to determine the risk factors associated with disease severity or mortality. If a certain blood group remains a risk factor for severe disease or mortality after a multivariate analysis, it would be more convincing to state that certain blood group is associated with severe disease and higher mortality.

Response: We re-evaluated the data and did multivariate analysis with adjusting comorbid conditions and age/gender

Issue 2: Abstract, conclusions, “…hospital stay, severity of disease, and mortality were associated with blood group A.” -> However, hospital stay was not different between all four blood groups as mentioned in the Result section “Patients with AB blood group stayed a median (IQR) of 14 (5, 27) days while A blood group cohort stayed 13 (6,27) days. It was statistically non-significant between all four groups.” In addition, the mortality rates among 4 blood groups were not statistically different according to Table 1.

Response: we corrected the abstract as recommended.plz see

Issue 3:  Results: “Regarding severity of COVID-19 disease, there was a trend towards critical disease in blood groups A and O (n=83, 41.1%; n=183, 35.1%; OR, 11.34 (95% CI, 46.79-53.22); p<0.001).” Why was OR ratio here beyond the range of 95% CI? In addition, to determine the effect of blood group A and O on disease severity, blood group A and O should have individual OR.

Response: OR corrected and added for both blood groups and mistakes rectified. Plz see

Issue 4: Discussion, “By contrast, we observed mild disease to be prevalent in the B and AB blood groups.“ -> The statement is not consistent with the data in Table 1.

Response: Very avid observation dear sir, we corrected the mistakes. Thank you for commenting. Plz see

Issue 5: Discussion, “Acute phase reactants were majorly deranged in blood group O as compared to other groups and hospital stay was associated more with A and AB blood groups”. -> However, according to Figure 2, blood group O appeared to have the highest levels of acute phase proteins, whereas blood group O had the lowest levels of acute phase proteins in Table 1. Please check these data again.

Response: we rechecked the data and new table is made with mean acute phae reactant values by our statistician. Plz see updated figure

Issue 6: Conclusions, “In conclusion, we demonstrate that male patients with blood group A and O are associated with an increased risk of severe COVID-19 disease after gender stratification while group A is associated with increased hospital stay and mortality.”-> According to the context, the effect of gender was not examined in this study. Furthermore, the association of group A with increased hospital stay and mortality was not evident according to Table 1.

Response: Thankyou dear sir for the observation. You are correct. We rectified the mistake

Minor comments:
1. Method, “The severity of COVID-19 was classified into, mild, moderate, and critical according to the Centers for Disease Control (CDC) guidelines.” -> Please provide reference here.
2. Please define the headings of Table 3 (B, SE, Wald).

Response: updated classification and reference added and table 2 headings dfined in table legend

Reviewer 2

Issue 1: I. In general, the English grammar should be checked before submission

Response: English syntax rechecked

Issue 2:  In the introduction, the authors should clearly describe the current status of studies about the association between ABO blood types and COVID-19. Both pros and cons options about this issue, especially severity and mortality, should also be cited in this section. Moreover, this section also lacks flow and transition sentences between paragraphs.

Response: updated investigations added to intro for a better clarity and syntax imporved

Issue 3: Materials and Methods
A. The definitions of COVID-19 severity are incorrect.
B. How to define COVID-19 related mortality?

Response: definition update for severity classification of covid 19, and any death in which no other cause was apparent other than covid 19 was considered as covid associated mortality

Issue 4: IV. Results
A. How about the distribution of comorbidities (other than those described by authors) between different blood types?
B. The therapeutic strategies between patients with different blood types should be pointed out.

Response: dear sit I regret to inform that no other comorbidities were collected in our study and no therapeutic strategy was noted. That data is not available to us unfortunately.

Issue 5:  Discussion
A. The second paragraph only describes the results of this study. No comparison was made with prior studies.
B. In the third paragraph, the authors described different prevalent results of blood types among various studies, including themselves. More discussion should be made about such issues.

Response: Discussion altered a little for more clarity

Issue 6; Tables and figures
A. Table 2 is not clear
B. The resolution of figures is poor

Figures cleared and table legends added

Editorial comments

Please include the tables within the main manuscript.

Included in the main text

Please note that in order to use the direct billing option the corresponding author must be affiliated with the chosen institute. Please either amend your manuscript to change the affiliation or corresponding author, or email us at plosone@plos.org with a request to remove this option.

Affiliation amended

Your ethics statement should only appear in the Methods section of your manuscript. If your ethics statement is written in any section besides the Methods, please move it to the Methods section and delete it from any other section. Please ensure that your ethics statement is included in your manuscript, as the ethics statement entered into the online submission form will not be published alongside your manuscript.

Ethics statement added to methods
