# Supplementary material for: Association of ABO blood group with COVID-19 severity, acute phase reactants and mortality
Source: PLoS One. 2021 Dec 14;16(12):e0261432. doi: 10.1371/journal.pone.0261432 (PMC8670663; doi:10.1371/journal.pone.0261432)
Supplement: Attachment — Submitted filename: response to reviewers.docx [file pone.0261432.s003.docx]

Dear editors and reviewers

Thank you for taking out time to comment on this manuscript. Your comments were valuable in making this manuscript better.

Point to point response

Abstract: “Acute phase protein reactants” was included in the manuscript title; however, nothing was mentioned in the abstract.

Response: abstract corrected and acute phase protein results added

Abstract (Method): Information provided in the method was very limited.

Response: method info increased as advised

Abstract (Results): “Patients with AB blood group stayed a median (IQR) of 14 (5, 27) days while A blood group cohort stayed 13 (6,27) days and overall…” It is difficult to understand why the authors mentioned days of hospital stay for AB blood group and A blood group here, as the days of hospital stay were not significantly different between 4 groups. Why was AB blood group mentioned here in particular?

Response: results amended. Please see

Abstract (Results): “…overall 10.6% COVID-19-related mortality was observed at our center, with 13.9% in blood group A as the majority of COVID-19 deaths.” This part could be written as a separate sentence. In addition, although patients of blood group A had a higher proportion of mortality, patients with blood group O contributed to the majority of deaths.

Response: statement amended

Abstract & Results: “Regarding severity of COVID-19 disease, there was a trend towards critical disease in blood group A (n=83, 41.1%; OR 0.257 (95% CI:0.167-1.136); p<0.001) and O (n=183, 35.1%; OR 0.438 (95% CI: 0.168-1.139); p<0.001).” The OR (95% CI) presented in this sentence were OR (95% CI) for “mortality” shown in Table 2 (multivariate analysis), but the proportions and p values presented in this sentence were the proportions and p values for critical diseases shown in Table 1 (univariate analysis). The presentation and interpretation of statistical results is confusing.

Response: p values corrected

“Multivariate analysis (after adjusting age, gender, and comorbidities) did not show any blood group in this cohort (Table 2).” This sentence was not clear.

Response: Sentence corrected and Multivariate of disease severity added in table 3

blood group O was linked to severe derangement of acute-phase reactants.” Data from Table 1 and Figure 2did not support this statement.

Response: all statements regarding acute phase reactants corrected please see

Alteration of the acute phase reactants is positively associated with blood type A.” Data from Table 1 and Figure 2 did not support this statement, and this statement was not consistent with “blood group O was linked to severe derangement of acute-phase reactants” mentioned in the Discussion section

Response: all statements regarding acute phase reactants corrected please see

Regards
