# Supplementary material for: Association of ABO blood group with COVID-19 severity, acute phase reactants and mortality
Source: PLoS One. 2021 Dec 14;16(12):e0261432. doi: 10.1371/journal.pone.0261432 (PMC8670663; doi:10.1371/journal.pone.0261432)
Supplement: Attachment — Submitted filename: response to reviewers.docx [file pone.0261432.s004.docx]

Dear editors and reviewers

Thank you for taking out time to comment on this manuscript. Your comments were valuable in making this manuscript better.

Point to point response

Question: In many instances, some minor differences existed between groups without statistical differences, but they were presented as meaningful findings.

Response: all minor differences corrected and meaningless findings excluded from the manuscript

Question: Some expressions are also confusing, for example, “the frequency of acute phase reactants with ABO blood type” in text and “Frequency distribution of acute phase reactants in correlation with ABO blood group” in Figure 2.

Response: all confusing statements corrected

Question:  In addition, the data of acute phase reactants in table 1 looked quite different with those in Figure 2.

Response: figure and table matched. Please see

Regards
