# Supplementary material for: Association of ABO blood group with COVID-19 severity, acute phase reactants and mortality
Source: PLoS One. 2021 Dec 14;16(12):e0261432. doi: 10.1371/journal.pone.0261432 (PMC8670663; doi:10.1371/journal.pone.0261432)
Supplement: Attachment — Submitted filename: response to reviewers.docx [file pone.0261432.s005.docx]

Dear editors and reviewers

Thank you for taking out time to comment on this manuscript. Your comments were valuable in making this manuscript better.

Point to point response

Question: Move sentence “Alteration of the acute phase reactants was not positively associated with any specific blood type” from section of Conclusion in Abstract to section of Results.

Response: Sentence moved to results

Question: It should be noted in the discussion that the outcome of patients' treatment was not examined in this study and this is one of the limitations of this study.

Response: Sentence added to limitations at the end of discussion

Question:  Check for spelling and typographical errors throughout the text.

Response: Errors fixed

Regards
